# Modeling of Pharmacokinetics of Cocaine in Human Reveals the Feasibility for Development of Enzyme Therapies for Drugs of Abuse

**DOI:** 10.1371/journal.pcbi.1002610

**Published:** 2012-07-26

**Authors:** Fang Zheng, Chang-Guo Zhan

**Affiliations:** Department of Pharmaceutical Sciences, College of Pharmacy, University of Kentucky, Lexington, Kentucky, United States of America; University of Warwick, United States of America

## Abstract

A promising strategy for drug abuse treatment is to accelerate the drug metabolism by administration of a drug-metabolizing enzyme. The question is how effectively an enzyme can actually prevent the drug from entering brain and producing physiological effects. In the present study, we have developed a pharmacokinetic model through a combined use of *in vitro* kinetic parameters and positron emission tomography data in human to examine the effects of a cocaine-metabolizing enzyme in plasma on the time course of cocaine in plasma and brain of human. Without an exogenous enzyme, cocaine half-lives in both brain and plasma are almost linearly dependent on the initial cocaine concentration in plasma. The threshold concentration of cocaine in brain required to produce physiological effects has been estimated to be 0.22±0.07 µM, and the threshold area under the cocaine concentration *versus* time curve (AUC) value in brain (denoted by AUC2_∞_) required to produce physiological effects has been estimated to be 7.9±2.7 µM·min. It has been demonstrated that administration of a cocaine hydrolase/esterase (CocH/CocE) can considerably decrease the cocaine half-lives in both brain and plasma, the peak cocaine concentration in brain, and the AUC2_∞_. The estimated maximum cocaine plasma concentration which a given concentration of drug-metabolizing enzyme can effectively prevent from entering brain and producing physiological effects can be used to guide future preclinical/clinical studies on cocaine-metabolizing enzymes. Understanding of drug-metabolizing enzymes is key to the science of pharmacokinetics. The general insights into the effects of a drug-metabolizing enzyme on drug kinetics in human should be valuable also in future development of enzyme therapies for other drugs of abuse.

## Author Summary

In this computational study, we have examined, for the first time, the potential effects of a drug-metabolizing enzyme on drug pharmacokinetics in human, showing that a high-activity drug-metabolizing enzyme can completely/effectively prevent the drug of abuse from entering brain to produce physiological effects. Based on this encouraging insight, it is feasible to develop enzyme therapies for drugs of abuse. Through pharmacokinetic modeling, we have demonstrated that, without an exogenous enzyme, the drug half-lives in both brain and plasma are almost linearly dependent on the initial drug concentration in plasma. This finding indicates that one may not simply say the half-life of a drug without clearly indicating the actual dose condition. We have also demonstrated for the first time how a high-activity drug-metabolizing enzyme can considerably decrease the peak concentration of drug in brain and drug half-lives in both brain and plasma. In addition, we have calculated the minimum (threshold) concentration of cocaine in brain required to produce physiological effects. The predicted threshold concentration, along with all of the general insights obtained in this study, will provide a rational base for future design of further experimental studies required for the enzyme therapy development.

## Introduction

Substance abuse and addiction are a major medical and social problem in the world [1]. Most of substances of abuse are psychoactive drugs, such as cocaine, illicit opiates, amphetamine-type stimulants, ecstasy-group substances, and cannabinoids. All psychoactive compounds have the abuse potential. Psychoactive drugs can cross the blood-brain barrier (BBB) and act primarily on the central nervous system (CNS) to alter brain functions, resulting in changes in perception, mood, consciousness, cognition, and behavior [2]. The disastrous social and economic consequences of drug abuse and addiction have made a high priority the anti-drug medication development.

Generally speaking, solving a drug addiction problem always needs to account for two aspects: antagonizing the stimulant effect of the abused drug, and bringing the function of brain's communication system back to normal. These two aspects are closely related to each other for an abused drug like cocaine which binds with dopamine transporter (DAT) in the same binding pocket as substrate dopamine. For example, cocaine addiction is associated with cocaine-induced change in the brain's communication system, including the rapid upregulation of DAT expression on the cell surface. One-time use of cocaine will increase the surface DAT expression for at least a month, as normalization of dopaminergic function is usually an extremely slow process [3]. Due to the increase of the surface DAT expression, there are less dopamine molecules available in the synapse for signaling, which likely contributes to the drug seeking or craving. So, it is necessary for therapeutic treatment of cocaine addiction to first (directly or indirectly) block the stimulant effects of cocaine. Without the stimulant effects of cocaine, one can have a real chance to bring the function of brain's communication system back to normal.

Pharmacological treatment of drug overdose and addiction can be either pharmacodynamic or pharmacokinetic [4]. Most of currently employed anti-addiction strategies use the classical pharmacodynamic approach. The classical pharmacodynamic approach aims to develop a small molecule that interacts with one or more neuronal binding sites, with the goal of blocking or counteracting neuropharmacological actions of the drug. However, due to the complex interrelations of neuronal circuits, it is difficult to accurately predict the actions of agonist/antagonist-type of therapeutic candidates and design an agonist/antagonist without undesirable side effects within the CNS [5]. In particular, as cocaine binds with DAT in the same binding pocket as dopamine [6], [7], [8], it would be extremely difficult to design an antagonist which can potently block DAT-cocaine binding without affecting the normal function of DAT. Hence, despite decades of effort [4], [9], there is still no FDA-approved therapeutic agent specific for cocaine. It is highly desirable to develop novel pharmacological approaches to treatment of cocaine overdose and addiction.

Due to the inherent difficulties in antagonizing a blocker like cocaine in the CNS, pharmacokinetic approaches are particularly interesting for possible treatment of cocaine overdose and addiction. A pharmacokinetic agent aims to act directly on the drug itself to alter its distribution or accelerate its clearance [4]. A pharmacokinetic agent could be a protein, such as an antibody, which binds tightly to the drug such that the drug-antibody complex cannot cross the BBB [4]. In practice, the anti-cocaine antibody could be provided with either active immunization (vaccine) or passive immunity (monoclonal antibody produced in another host). A pharmacokinetic agent could also be an appropriately designed cocaine-metabolizing enzyme [10], [11] that not only binds with cocaine but also accelerates cocaine metabolism and thereby freeing itself for further binding. Thus, an enzyme molecule can be used repeatedly until all cocaine molecules are metabolized. Clearly, a pharmacokinetic approach (enzyme, antibody or vaccine) has a potential advantage over the classic pharmacodynamic approach using a small-molecule agonist/antagonist, because a pharmacokinetic agent usually does not cross the BBB to reach the CNS. So, an appropriately designed pharmacokinetic agent is not expected to block the normal functions of the transporters and receptors in the CNS [10]. Concerning the enzyme-based approach, the remaining question is how effective a drug-metabolizing enzyme can prevent the drug from entering brain and producing physiological effects. Here we address this question by taking cocaine pharmacokinetics [12], [13] as a typical example.

It has been recognized as a promising anti-cocaine medication to accelerate cocaine metabolism in plasma, producing biologically inactive metabolites *via* a route similar to the primary cocaine-metabolizing pathway in plasma, *i.e.* cocaine hydrolysis catalyzed by butyrylcholinesterase (BChE), as BChE-catalyzed hydrolysis of cocaine produces biologically inactive metabolites [9], [10]. The other possibly valuable pharmacokinetic agents capable of accelerating cocaine metabolism that have been investigated so far include anti-cocaine catalytic antibodies [11], [14], [15] and bacterial cocaine esterase (CocE) [16], [17], [18]. The catalytic antibodies, wild-type BChE, and BChE mutants discovered earlier have a low catalytic activity against naturally occurring (−)-cocaine [19], [20], [21], [22], [23]. However, CocE (particularly its thermostable mutants) [24] and our recently designed mutants of human BChE with a considerably improved catalytic activity against (−)-cocaine [9], [25], [26], [27], [28], [29], [30], [31] are promising for not only therapeutic treatment of cocaine overdose [29], [32], [33], but also possible treatment of cocaine abuse [33], [34]. It has also been demonstrated that our rationally designed mutations on human BChE only considerably and selectively increase the catalytic activity of the enzyme against (−)-cocaine without a significant increase in the catalytic activities against other substrates tested [32], including acetylcholine (which is the only natural substrate in the body), suggesting that these BChE mutants are safe. Our reported BChE mutants with at least ∼1,000-fold improved catalytic activity against (−)-cocaine are also known as cocaine hydrolases (CocHs) in literature [35].

Strategy using an enzyme to treat cocaine abuse is based on the idea that the enzyme can alter the pharmacokinetics of cocaine in a favorable manner by rapidly metabolizing cocaine. In this way, an enzyme agent could reduce cocaine's entry into the brain to a certain amount (threshold) that is too low to cause cocaine-elicited drug seeking or toxicity *etc.* [9] Plenty of experimental results from animal studies have demonstrated that an enzyme like CocH or CocE with a high catalytic activity against (−)-cocaine is effective to protect animals from the acute cocaine toxicity [23], [30], [32], [33], [34], [36]. In addition, our previously reported A199S/S287G/A328W/Y332G mutant of human BChE (denoted by CocH1 in this report for convenience) [28] was fused with human serum albumin by Brimijoin *et al.* [36] to extend its *in vivo* half-life. It has been shown that the albumin-fused CocH1 and thermosatble CocE mutants are all capable of reducing the stimulant effects of the self-administered cocaine and blocking cocaine-primed reinstatement of drug-seeking [33], [34], [36]. For the treatment of cocaine abuse, one would expect that a CocH or CocE can be used to build a strong defense system through rapidly degrading cocaine in plasma so that the cocaine molecules are prevented from entering the brain.

Therefore, it is important to understand cocaine pharmacokinetics in the presence of a high-activity cocaine hydrolase. For the purpose, we first theoretically evaluated CocH as a potential therapeutic treatment of cocaine overdose and abuse. The evaluation is based on the development of a two-compartment pharmacokinetic model (representing the time-dependent concentrations of (−)-cocaine in plasma and in brain) with Michaelis-Menten elimination of cocaine in plasma of human (*i.e.* the endpoint user of our CocH). Pharmacokinetic modeling can be performed to appropriately address such essential questions as: What amount of cocaine can a given concentration of the cocaine-metabolizing enzyme effectively prevent from entering brain and producing physiological effects? What are the minimum requirements for the catalytic activity and concentration of the enzyme used to treat a given dose of cocaine? What are the effects of the catalytic parameters (catalytic rate constant *k*_cat_ and Michaelis-Menten constant *K*_M_) of the enzyme on the cocaine abuse treatment? Insights obtained from the pharmacokinetic modeling could provide a clear roadmap and baseline for future development of an enzyme-based therapy for cocaine abuse. Such pharmacokinetic modeling would help to make the *in vivo* studies much more efficient and, thus, help to considerably reduce the animal sacrifice, experimental labors, and financial expenditure for the *in vivo* tests with the enzyme and cocaine. In the present study, we have developed a pharmacokinetic model by utilizing available experimental data from the direct measurements of cocaine in the brains and plasma of normal human volunteers made by using positron emission tomography (PET) and tracer doses of [^11^C]cocaine, *i.e.* N-^11^C-methyl-(-)-cocaine [37]. The developed pharmacokinetic model has been used to clearly address the fundamental questions mentioned above, for the first time, providing valuable insights into the effects of the enzyme on the action of cocaine.

## Methods

### Theoretical model

**Figure 1:**
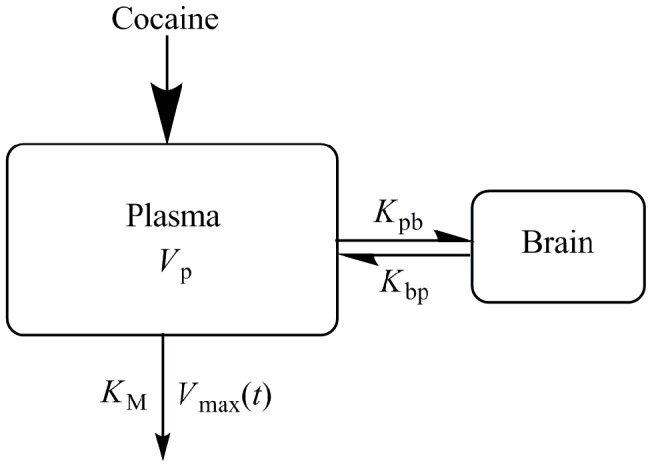
A two-compartment model. The model has one compartment representing brain (striatum) tissue, which exchanges radiotracer with plasma compartment (volume *V*_p_) with two diffusion constants *K*_pb_ and *K*_bp_. In the plasma compartment, cocaine molecules experience an enzymatic Michaelis-Menten elimination, where *K*_M_ is the Michaelis-Menten constant and *V*_max_ is the maximum velocity of cocaine conversion to the metabolites.

A two-compartment *in vivo* pharmacokinetic model (Figure 1) with Michaelis-Menten elimination in plasma was built to describe the cocaine concentration-time profile in the human body and characterize the relationship between the profile and the observed pharmacological activity of cocaine reported by Fowler *et al.* [37] Assuming that all compartments are homogeneous, the model is based on the following differential equations:
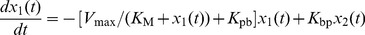
(1)

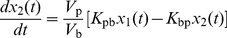
(2)where 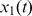 and 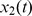 represent the cocaine concentrations in plasma (compartment 1) and brain (compartment 2), respectively. *K*_M_ is the Michaelis-Menten constant of the enzyme for cocaine. *V*_max_ is the maximum rate of the enzymatic reaction when the enzyme is saturated by the substrate (cocaine): *V*_max_≡*k*_cat_·[E] in which *k*_cat_ is the catalytic rate constant and [E] is the concentration of the enzyme in plasma. *K*_pb_ is the constant for cocaine diffusion from plasma compartment to brain compartment, and *K*_bp_ is the constant for cocaine diffusion from brain compartment to plasma compartment. *V*_p_ and *V*_b_ refer to the effective volumes of compartments 1 (plasma) and 2 (brain), respectively. Standard Michaelis-Menten equation of BChE-catalyzed cocaine hydrolysis was used for a couple of reasons: (1) BChE-catalyzed hydrolysis of cocaine is the dominant cocaine-metabolizing pathway in plasma and the other cocaine-metabolizing pathways may be neglected [10]; (2) the products of BChE-catalyzed hydrolysis of cocaine do not significantly inhibit BChE [38]. Further, in the presence of an exogenous cocaine-metabolizing enzyme with a >1,000-fold improved catalytic efficiency against cocaine, the catalytic activity of the endogenous enzyme is negligible.

Concerning the structural identifiability [39], [40], [41] of the model, there are two model outputs, denoted by 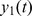 and 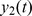 for convenience, and we have 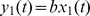 and 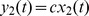 in which 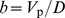 as discussed below. *V*_b_, *V*_p_, *K*_pb_, and *K*_bp_ are unknown parameters/variables, whereas *c*, *V*_max_, and *K*_M_ are the known constants (see below). It should be noted that *V*_b_, *V*_p_, *K*_pb_, and *K*_bp_ all must be positive values in order to be physically meaningful. An analysis using the Taylor series expansion method revealed that there is only one physically meaningful solution for the values of parameters 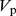, 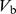, 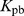, and 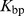 used in the model. So, under the condition that 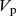, 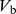, 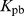, and 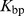 all must be positive values, all unknown parameters of the model associated with Eqs.(1) and (2) are uniquely identifiable and, therefore, the model is structurally identifiable.

### Model fitting and parameter estimation

PET data were selected to fit the model as PET is superior over other techniques, such as microdialysis, that have been used to determine the cocaine distribution and its time course in living subject (human). In addition, PET imaging analysis can reveal the variation of cocaine concentration in brain starting from seconds after cocaine is injected into a living subject. Therefore, the theoretical model was fitted to the experimentally observed data reported by Fowler *et al.* [37] These PET data were chosen based on several reasons. First, the PET data were obtained for human subjects, which is consistent with *k*_cat_ and *K*_M_ of human BChE that we have been studying in our lab [28], [29], [30], [31], [32]. Second, the cocaine concentrations in plasma at various time points were also measured for the same subject(s) along with the PET measurement. In addition, the time course of cocaine appearing in the striatum of brain [37] is consistent with the time course of the mean subjective “high” in human subjects reported by Cook *et al.*, [42] a well-documented euphoria experienced after the intravenous (i.v.) administration of cocaine.

**Figure 2:**
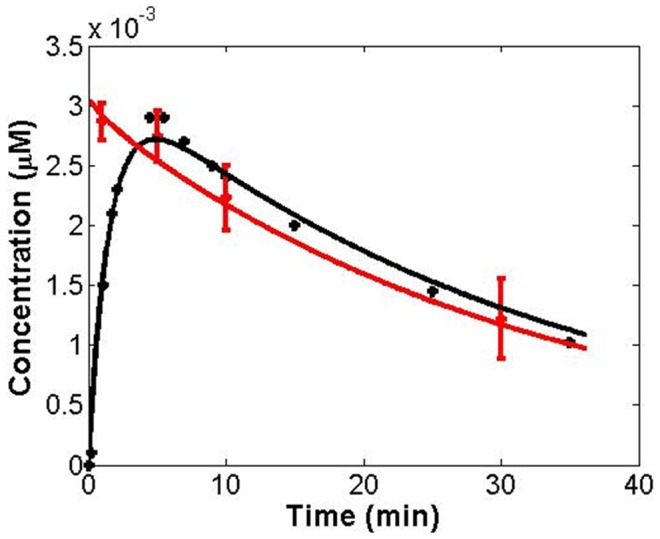
Modeling of available experimental data (dots) for the concentrations of cocaine in brain (black) and in plasma (red) of human subject. The experimental data came from reference [37].

The experimental data – uptake and clearance of the [^11^C]cocaine radioactivity in the human corpus striatum over a 35-minute period after the injection of [^11^C]cocaine depicted in Figure 2 of the previous report [37], and the relative concentration of [^11^C]cocaine in human plasma depicted in Figure 4A of the previous report [37] – were digitized for fitting with the results obtained from the theoretical pharmacokinetic simulation. Although the experimental data for uptake and clearance of the [^11^C]cocaine radioactivity in the human cerebellum were also available, we chose not to directly model these data in our finally generated model because cocaine always had the highest concentration in the striatum [37], [43]. To include the uptake and clearance of radioactivity in the human cerebellum, one more compartment and three additional parameters would need to be included in the pharmacokinetic model. The extra parameters would add some additional flexibility (and also uncertainty) to the model during the calibration of the model parameters. The difference for the distribution of (−)-cocaine in brain other than striatum can be corrected by adjusting the volume parameter *V*_b_ during the model fitting.

Our pharmacokinetic modeling and simulation were performed by use of ADAPT II program [44] and a MATLAB program developed in our own lab for numerical solution of differential equations defined in Eqs.(1) and (2) [45], [46], [47]. Curves of the cocaine concentrations *versus* time in both compartments generated by numerical integration were evaluated for the closest to the observed PET data. The fitting was judged by using the root-mean-squared error (RMSE). All points were given the equal weight during the least-squares fitting process.

### Area under the curve (AUC) analysis

The cocaine exposure in brain can be characterized by the area under the cocaine concentration *versus* time curve (AUC) in brain. For convenience, the AUC values in plasma and brain within time *t* after the cocaine administration are denoted by AUC1*_t_* and AUC2*_t_*, respectively, and can be evaluated *via*

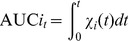
(3)in which *i* = 1 and 2 that refer to plasma and brain, respectively. In practice, the numerical integration with Eq.(3) was carried out by using the well-known linear trapezoidal rule. AUC*i_t_* = AUC*i*_∞_ when *t* = ∞.

## Results

### Model parameters

We have developed a two-compartment *in vivo* pharmacokinetic model. The model has compartments: one compartment represents brain (striatum) tissue with volume *V*_b_, and the other represents plasma with volume *V*_p_. The compartments exchange cocaine molecules, characterized by two diffusion constants: *K*_pb_ (for cocaine diffusion from plasma to brain) and *K*_bp_ (for cocaine diffusion from brain to plasma). In the plasma compartment, cocaine molecules experience an enzymatic Michaelis-Menten elimination, where [E] is the enzyme concentration, *K*_M_ is the Michaelis-Menten constant, and *k*_cat_ is the catalytic rate constant. The model based on Eqs.(1) and (2) involves the following variables: *k*_cat_, *K*_M_, [E], *V*_b_, *V*_p_, *K*_pb_, and *K*_bp_. Endogenous BChE in human plasma primarily exists in tetramer with a mass of 340 kDa. The tetramer accounts for 95% BChE; the remaining 5% exists in dimer (170 kDa) and monomer (85 kDa) [48]. All of these protein oligomers are active forms of BChE. A monomer has one active site. A dimer has two active sites. However, in the tetramer structure, only two subunits have their catalytic sites accessible to the substrate [49], [50], while the catalytic sites of the other two subunits are blocked by the neighboring subunits. Thus, a tetramer of BChE only has two truly active sites. For this reason, when we discuss the concentration and catalytic activity of BChE, it is more convenient to talk about the molar concentration of the BChE active site. So, the enzyme concentration [E] mentioned below will always refer to the molar concentration of the BChE active site. It has been known that for the endogenous BChE in normal human plasma, *k*_cat_ = 4.1 min^−1^, *K*_M_ = 4.5 µM based on *in vitro* activity assays [21], and the enzyme concentration can be taken as [E] = 0.035 µM (a constant) which is close to the medium value within the reference concentration range (4.6 to 14 KU/L) established in humans for BChE [51]. Based on these known *k*_cat_, *K*_M_, and [E] values, the other parameters, including *V*_b_, *V*_p_, *K*_pb_, and *K*_bp_, can be determined/optimized by fitting to the experimental data [37] concerning 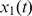 and 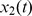 *versus* time in plasma and brain, respectively.

Parameters *K*_bp_ and *K*_pb_ determine how fast cocaine diffuses between brain and plasma. By definition, *V*_b_ and *V*_p_ are the effective volumes of brain and plasma, respectively. Concerning *V*_p_, a typical adult has a blood volume between 4.7 and 5 L including ∼3 L plasma. It seems reasonable to assume that *V*_p_ is about ∼3 L. However, cocaine can actually exist in other parts of the body in addition to plasma and brain. As a result, a reasonable (effective) *V*_p_ value should be significantly larger than 3 L. Further, for the given experimental dose of [^11^C]cocaine at ∼11 µg [37], the used *V*_p_ value determines the initial concentration of [^11^C]cocaine (when *t* = 0) in plasma. Hence, in principle, the *V*_p_ value may be evaluated by using the measured 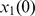 value, *i.e.* the initial concentration of [^11^C]cocaine in plasma. However, it has been difficult to determine 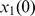 accurately in human plasma. Reported in ref. [37] were the relative 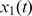 values, or 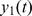 values with 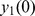 = 1 (*i.e.* 100%). So, *V*_p_ was regarded as an adjustable parameter. Because 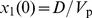 and 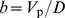 (in which *D* is the dose of cocaine used), once *V*_p_ is known, the *b* and 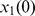 values are all known. For the similar reason, concerning the amount of the overall cocaine entry to brain compartment, *V*_b_ was also considered as an adjustable parameter which should be between the volume of striatum alone (6.33±0.44 cm^3^) and that of the whole brain (1,253.8±70.90 cm^3^) [52].

The *V*_b_, *V*_p_, *K*_pb_, and *K*_bp_ values were optimized according to three criteria. The first is the RMSE for the 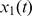 values, denoted by RMSE1; the smaller the RMSE values, the better the model. The second is the RMSE for the 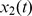 values, denoted by RMSE2; the smaller the RMSE values, the better the fitting. In addition, the ratio of AUC2*_∞_* to AUC1*_∞_* should be consistent with available experimental observations. Through microdialysis studies on freely-moving rats, Hedaya *et al.* [53], [54] observed that the ratio of cocaine AUC in brain extracellular fluid to that in plasma after the cocaine administration was 1.20±0.18 (or 1.02 to 1.38). In light of their experimental results, the ratio of AUC2*_∞_* to AUC1*_∞_* is expected to be slightly larger than 1 within the range of 1.02 to 1.38. Based on the fitting, we obtained *V*_b_ = 0.3292 L, *V*_p_ = 11.89 L, *K*_pb_ = 0.01898 min^−1^, and *K*_bp_ = 0.01780 min^−1^. The fitted curves are depicted in Figure 2. The corresponding computational and experimental values are provided as supporting information for comparison. By using the optimized *V*_b_, *V*_p_, *K*_pb_, and *K*_bp_ values, we obtained RMSE1 = 0.00010 µM with Pearson correlation coefficient *r* = 0.992, RMSE2 = 0.000128 µM with *r* = 0.995, AUC1*_∞_* = 0.091 µM·min, and AUC2*_∞_* = 0.097 µM·min. The data depicted in Figure 2, the low RMSE values, and the high Pearson correlation coefficient values all suggest that the fitting was satisfactory.

The optimized *V*_b_ value of 0.3292 L is within the aforementioned range between the volume of striatum alone (6.33±0.44 cm^3^) and that of the whole brain (1,253.8±70.90 cm^3^) as expected. The optimized *V*_p_ value of 11.89 L is larger than the typical volume (∼3 L) of the plasma, as expected in the above discussion of the possible reasons. The difference between the optimized *V*_p_ value and the typical plasma volume may be also partially due to the possible errors of other non-adjustable parameters ([E], *k*_cat_, and *K*_M_). In particular, the used *k*_cat_ and *K*_M_ values were determined *in vitro* as mentioned above, and the actual *in vivo* activity of the endogenous enzyme (wtBChE) could deviate from the measured *in vitro* activity of the same enzyme. Further, based on the optimized *K*_pb_, *K*_bp_, AUC1*_∞_*, and AUC2*_∞_* values, we have AUC2*_∞_*/AUC1*_∞_*≈*K*_pb_/*K*_bp_ = ∼1.1. The result of AUC2*_∞_*/AUC1*_∞_*≈*K*_pb_/*K*_bp_ and the well-fitted curves depicted in Figure 2 also suggest that cocaine diffusion across the BBB was fast enough to always keep an equilibrium distribution between brain and plasma after a few minutes. As well known, like many other amine drugs, a cocaine molecule has two protonation states (*i.e.* the protonated cocaine which is the active cocaine state and the free base which can easily cross the BBB) coexisting in the body *via* rapid protonation and deprotonation processes. Whereas the protonated cocaine state is responsible for its biological function, the free base state is responsible for crossing the BBB. The two states of cocaine can quickly reach the thermodynamic equilibrium associated with p*K*_a_ = 8.6 [55]. One may expect that the *K*_pb_/*K*_bp_ value should be close to the ideal value 1.0 for the diffusion process. The optimized *K*_pb_/*K*_bp_ value of ∼1.1 is slightly larger than 1.0, which might be attributed to the implicitly used approximation which ignores DAT binding with cocaine in the brain. Nevertheless, the minor difference between the optimized *K*_pb_/*K*_bp_ value of ∼1.1 and the ideal value 1.0 suggests that the effect of the DAT binding is not dramatic. The result of AUC2*_∞_*/AUC1*_∞_* = ∼1.1 is consistent with the above-mentioned range (1.02 to 1.38), which further suggests that the model is reasonable. The reasonable model has been used to predict cocaine concentrations in brain under various dose conditions (with [E] being a constant), as discussed below. See supporting information for more detailed data and discussion of the impacts of the catalytic parameters of enzyme on the cocaine concentration in brain. [E] was considered as a constant for all enzymes in the present study, because it was demonstrated [56] that the constant enzyme concentration can be reached through delivering the genes of the enzyme (corresponding to the enzyme-based gene therapy). The cocaine hydrolase gene therapy aims to produce a constant enzyme concentration in plasma. The reported gene delivery of a BChE mutant successfully produced the BChE mutant in rat plasma with a concentration as high as 0.5 µM [56].

### Correlation between the initial cocaine concentration in plasma and cocaine half-life in brain

Now that the values of all parameters (*i.e. k*_cat_, *K*_M_, [E], *V*_b_, *V*_p_, *K*_pb_, and *K*_bp_) in Eqs.(1) and (2) have been known, the model can be used to examine whether cocaine half-life in brain is dependent on the initial dose of cocaine in plasma, *i.e.* 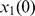. Here, the cocaine half-life in brain, denoted by *t*_b1/2_, is defined as the time (*t*) when 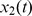 is equal to a half of the peak concentration of cocaine, *i.e.* the maximum 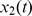 value, in brain. *t*_b1/2_ becomes longer when 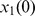 increases, which indicates the nonlinearity of cocaine pharmacokinetics (see supporting information for the plots). A drug with nonlinear pharmacokinetics has a half-life that varies as a function of the (initial) cocaine plasma concentration. With only wild-type BChE (wtBChE), *t*_b1/2_ increases with 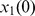 almost linearly. When *t*_b1/2_ is in min and 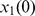 is in µM, the linear variation of cocaine half-life in brain can be described by an approximate equation:
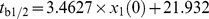
(4)with a high correlation coefficient (*r*) of 0.9998. Accordingly, the linear variation of cocaine half-life in plasma can be represented as
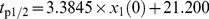
(5)with *r* = 1.0000. Eqs.(4) and (5) were obtained from fitting a straight line model to the cocaine half-lives predicted actually from the pharmacokinetic model. Equations (4) and (5) clearly reveal that the cocaine half-lives in both plasma and brain are linearly dependent on the actual cocaine dose which determines the initial cocaine concentration in plasma, *i.e.* the 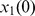 value in the equations. The approximate linear correction reflected in Eqs.(4) and (5) can be attributed to the saturation of the endogenous cocaine-metabolizing enzyme (wtBChE) when the cocaine concentration is high compared to the rather low concentration (0.035 µM) of wtBChE with *K*_M_ = 4.5 µM. When the enzyme is saturated, further increasing the initial cocaine concentration can no longer increase the overall velocity of the cocaine metabolism and, therefore, the time required to metabolize a half amount of cocaine will be linearly proportional to the initial concentration of cocaine. Certainly, when the initial cocaine concentration is very low compared to the corresponding concentration of the endogenous enzyme, the actual correlation between the cocaine half-life and the initial cocaine concentration will significantly deviate from the ideal linear correction.

We note that, in theory, the half-life of a drug in nonlinear kinetics is not a constant because the rate of elimination is dependent on the drug concentration. Nevertheless, *in vivo* half-lives of cocaine have been reported in literature and, thus, Eqs.(4) and (5) may be used conveniently to better understand and predict the experimental half-lives. In addition, under the same cocaine dose condition, the relative half-lives of cocaine reflect the relative *in vivo* potency of various cocaine-metabolizing enzymes. Notably, the linear dependence of cocaine half-life in plasma is qualitatively consistent with the experimental observation reported by Barntt *et al.* over the cocaine dose range of 1–3 mg/kg when cocaine was given to human subjects intravenously [57].

In the presence of CocH3 (*i.e.* A199S/F227A/S287G/A328W/Y332G mutant of human BChE) with a *K*_M_ = 3.1 µM and *k*_cat_ = 5,700 min^−1^) [30], when 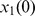
<200 µM, cocaine half-life, *t*_b1/2_, in brain is almost a constant of 1.1 min; only in the case of extremely high cocaine concentration when 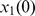
>200 µM, *t*_b1/2_ increases slightly; see supporting information for the plots of *t*_b1/2_ *versus* 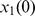. The enzyme-caused remarkable decrease in *t*_b1/2_ clearly suggests that CocH3 is an excellent agent for protecting a living subject from the cocaine toxicity, which has been verified by the fact that a small dose (0.01 mg/mouse, i.v.) of CocH3 can fully protect the mice from the acute toxicity of a lethal dose of cocaine (180 mg/kg, i.p.) [30], [32]. The encouraging results with CocH3 reveal that increasing the catalytic activity of the cocaine-metabolizing enzyme in plasma indeed can decrease the half-life (*t*_b1/2_) of cocaine in brain.

### Evaluation of available high-activity cocaine-metabolizing enzymes for their effects on the cocaine concentration in brain

The calibrated model has been employed to evaluate currently available high-activity cocaine-metabolizing enzymes, including CocE, CocH1 (*i.e.* A199S/S287G/A328W/Y332G mutant of human BChE), CocH2 (*i.e.* A328W/A199S/F227A/E441D/S287G mutant of human BChE), and CocH3 (*i.e.* A199S/F227A/S287G/A328W/Y332G mutant of human BChE), for their effects on the cocaine concentration in brain with a given initial cocaine concentration in plasma. According to the previous reports [17], [18], the catalytic efficiency (*k*_cat_/*K*_M_) of wild-type bacterial CocE (*k*_cat_ = 468 min^−1^ and *K*_M_ = 0.64 µM) against (−)-cocaine is ∼800 fold higher than that of wild-type human BChE. CocH1 (*k*_cat_ = 3,060 min^−1^ and *K*_M_ = 3.1 µM) [29], [31], [32], CocH2 (*k*_cat_ = 1,730 min^−1^ and *K*_M_ = 1.1 µM) [29], [31], [32], and CocH3 (*k*_cat_ = 5,700 min^−1^ and *K*_M_ = 3.1 µM) [29], [30], [31], [32] has a ∼1,080-, ∼1,730-, and ∼2,020-fold improved catalytic efficiency, respectively, compared to wild-type human BChE against (−)-cocaine.

**Figure 3:**
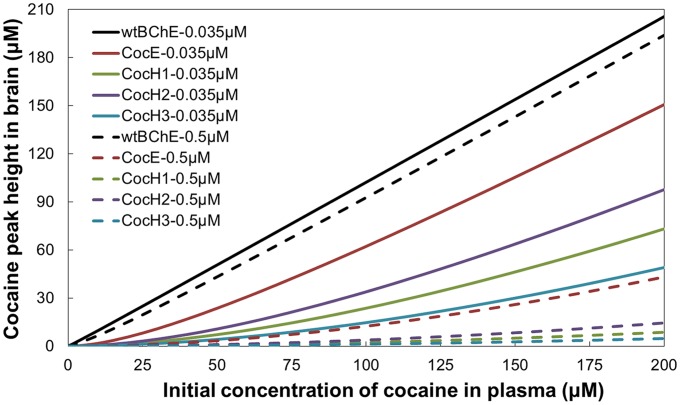
Plots for the cocaine peak concentration (µM) in brain *versus* the initial concentration (µM) of cocaine in plasma in the presence of a cocaine-metabolizing enzyme (0.035 or 0.5 µM).

With each of these high-activity enzymes, we examined their effects under various concentrations. Two enzyme concentrations were examined: 0.035 and 0.5 µM. We are interested in 0.035 µM, because it is the physiologic concentration of the endogenous BChE (wtBChE) as discussed above. We are also interested in 0.5 µM, because it has been known that a gene transfer produced a BChE mutant with a constant concentration of 0.5 µM in plasma for a few months [56], [58]. According to previous studies [23], [59], the most interesting cocaine doses relevant to the addiction and overdose are associated with plasma cocaine concentrations between 0.32 µM and 200 µM. 1–5 µM cocaine in plasma are regarded as the commonly abused doses to be used to get the reward effects of cocaine [60], whereas 3–200 µM cocaine in plasma would be considered as an overdose with the higher end being lethal [23]. Thus, we examined 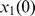 = 0 to 200 µM. Numerical results of the cocaine peak concentration, the peak time, *t*_b1/2_, and the area under curve in human brain (AUC2_∞_) obtained for four exogenous enzymes with five representative 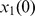 values (1, 5, 50, 100, and 200 µM) corresponding to [E] = 0.035 µM are summarized in Table 1. The corresponding results corresponding to [E] = 0.5 µM are provided in supporting information. Depicted in Figure 3 are the plots of the peak concentration of cocaine in brain *versus* 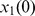 for the four enzymes with [E] = 0.035 or 0.5 µM in plasma.

**Table 1:** Lookup table for the predicted cocaine peak concentration, the peak time (Ptime in min), *t*_b1/2_ (in min), and the area under curve (AUC2_∞_ in human brain for a given initial concentration of cocaine with CocE or CocH or endogenous wtBChE in plasma ([E] = 0.035 µM).

COC	wtBChE		CocE		CocH1		CocH2		CocH3	
(µM)	(*K_M_* = 4.5, *k_cat_* = 4.1)		(*K_M_* = 0.64, *k_cat_* = 468)		(*K_M_* = 3.1, *k_cat_* = 3060)		(*K_M_* = 1.1, *k_cat_* = 1730)		(*K_M_* = 3.1, *k_cat_* = 5700)	
	Peak	AUC2_∞_	Peak	AUC2_∞_	Peak	AUC2_∞_	Peak	AUC2_∞_	Peak	AUC2_∞_
	(Ptime)		(Ptime)		(Ptime)		(Ptime)		(Ptime)	
	[t_b1/2_]		[t_b1/2_]		[t_b1/2_]		[t_b1/2_]		[t_b1/2_]	
1	0.908	35.101	0.043	0.074	0.021	0.036	0.017	0.028	0.012	0.019
	(5.101)		(0.187)		(0.122)		(0.091)		(0.075)	
	[27.006]		[1.140]		[1.088]		[1.119]		[1.096]	
5	4.738	257.458	0.547	1.019	0.164	0.279	0.189	0.317	0.091	0.150
	(5.833)		(0.372)		(0.147)		(0.140)		(0.088)	
	[39.863]		[1.110]		[1.102]		[1.109]		[1.083]	
50	50.649	10694.160	23.636	81.804	7.204	13.917	10.732	22.789	4.239	7.488
	(8.401)		(1.677)		(0.471)		(0.683)		(0.264)	
	[193.631]		[1.868]		[1.128]		[1.206]		[1.102]	
100	102.245	39431.860	62.272	321.378	23.679	52.378	33.898	88.718	14.701	28.224
	(9.337)		(2.455)		(0.782)		(1.135)		(0.471)	
	[366.658]		[3.190]		[1.206]		[1.362]		[1.128]	
200	205.639	151038.500	150.715	1274.122	73.174	202.176	97.659	348.698	49.046	109.120
	(10.380)		(3.350)		(1.249)		(1.768)		(0.782)	
	[715.647]		[5.953]		[1.440]		[1.945]		[1.206]	

The *K*_M_ values are given in µM, the *k*_cat_ values are given in min^−1^, and the AUC2_∞_ values are given in µM·min.

A survey of the data in Table 1 reveals that the use of a high-activity enzyme (CocH or CocE) and/or the increase of the enzyme concentration all can decrease AUC2*_∞_*, *t*_b1/2_, peak cocaine concentration, and peak time in brain. Because AUC2*_∞_* and cocaine peak concentration in brain are the primary determinants of the overall cocaine reward/stimulation effects in brain, CocHs and CocE all can considerably decrease the overall cocaine reward/stimulation effects in brain.

### Threshold concentration of cocaine in brain to produce physiological effects

For development of a CocH-based therapy for cocaine abuse, it is essential to know whether a cocaine-metabolizing enzyme can effectively prevent cocaine from entering brain and producing physiological effects. When the cocaine concentration in brain has never reached a “threshold” value in the presence of a CocH in plasma, one may consider that the CocH has *effectively* prevented cocaine from entering brain. Further, the threshold concentration of cocaine in brain is related to the degree of the dopamine transporter (DAT) occupancy by cocaine. Volkow and Fowler *et al.* demonstrated that, for humans, “at least 47% of the transporters had to be blocked for subjects to perceive cocaine's effects” and that 0.1 mg/kg cocaine (i.v.) producing 97±25 ng/ml (∼0.32 µM) cocaine in plasma (the peak concentration) blocked 47% of the transporters [59]. In comparison, 0.05 mg/kg cocaine (i.v.) blocked only 41% of the transporters and, thus, had no measurable effects [59]. These experimental data for human subjects in combination with our pharmacokinetic modeling have provided an opportunity to estimate the threshold concentration of cocaine in brain required to produce measurable physiological effects.

With only the endogenous BChE at the normal concentration (0.035 µM) in human plasma, when 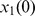 = 0.32 µM (associated with the 0.1 mg/kg cocaine administered i.v.), the peak concentration of cocaine in brain is calculated to be 0.29 µM and AUC2*_∞_* = 10.5 µM·min. For comparison, when 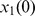 = 0.16 µM (associated with the 0.05 mg/kg cocaine administered i.v.), the peak concentration of cocaine in brain is calculated to be 0.14 µM and AUC2*_∞_* = 5.2 µM·min. Based on these results, one can estimate that the threshold concentration of cocaine in brain to produce measurable physiological effects should be between 0.14 and 0.29 µM (or 0.22±0.07 µM), and that the threshold AUC2*_∞_* value of cocaine in brain to produce measurable physiological effects should be between 5.2 and 10.5 µM·min (or 7.9±2.7 µM·min).

According to the estimated threshold brain cocaine concentration of ∼0.22 µM, the pharmacokinetic modeling can be performed to predict/estimate how much cocaine a given concentration of cocaine-metabolizing enzyme can effectively prevent from entering brain and producing physiological effects (see Table 2 for the predictions/estimates). For example, in the presence of 0.035 µM CocH3, the peak brain cocaine concentration corresponding to 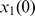 = 8 µM is predicted to be lower than ∼0.22 µM. Hence, we may say that 0.035 µM CocH3 can effectively prevent up to ∼8 µM cocaine in plasma from entering brain and producing physiological effects. 0.5 µM CocH3 can effectively prevent up to ∼40 µM cocaine in plasma from entering brain and producing physiological effects.

**Table 2:** The maximum cocaine plasma concentration, *i.e.* the 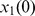 value (µM), which a given concentration ([E] = 0.035 or 0.5 µM) of cocaine-metabolizing enzyme (CocE, CocH1, CocH2, or CocH3) can effectively prevent from entering brain and producing physiological effects.

[E]	Criterion	CocE	CocH1	CocH2	CocH3
0.035 µM	Threshold peak^a^	2	6	5	8
	Threshold AUC2_∞_ b	15	35	28	50
0.5 µM	Threshold peak^a^	11	28	22	40
	Threshold AUC2_∞_ b	55	145	110	200

a The criterion used is the threshold peak cocaine concentration of ∼0.22 µM in brain.

b The criterion used is the threshold AUC2*_∞_* value of ∼7.9 µM·min in brain.

As seen in Table 2, the theoretical predictions based on the threshold AUC2*_∞_* value are significantly different from the corresponding predictions based on the threshold peak cocaine concentration in brain. For example, according to the estimated threshold AUC2*_∞_* value of ∼7.9 µM·min and the pharmacokinetic modeling, one may also predict/estimate that 0.035 µM CocH3 can effectively prevent up to ∼50 µM cocaine in plasma from entering brain and producing physiological effects, and 0.5 µM CocH3 can effectively prevent up to ∼200 µM cocaine in plasma from entering brain and producing physiological effects. It is reasonable to expect that the above predictions based on the threshold peak brain cocaine concentration of ∼0.22 µM provide the lower limits, whereas the corresponding predictions based on the threshold AUC2*_∞_* value of ∼7.9 µM·min give the upper limits.

### Comparison between the catalytic activities of CocH3 against (−)-cocaine and native BChE against (+)-cocaine

**Figure 4:**
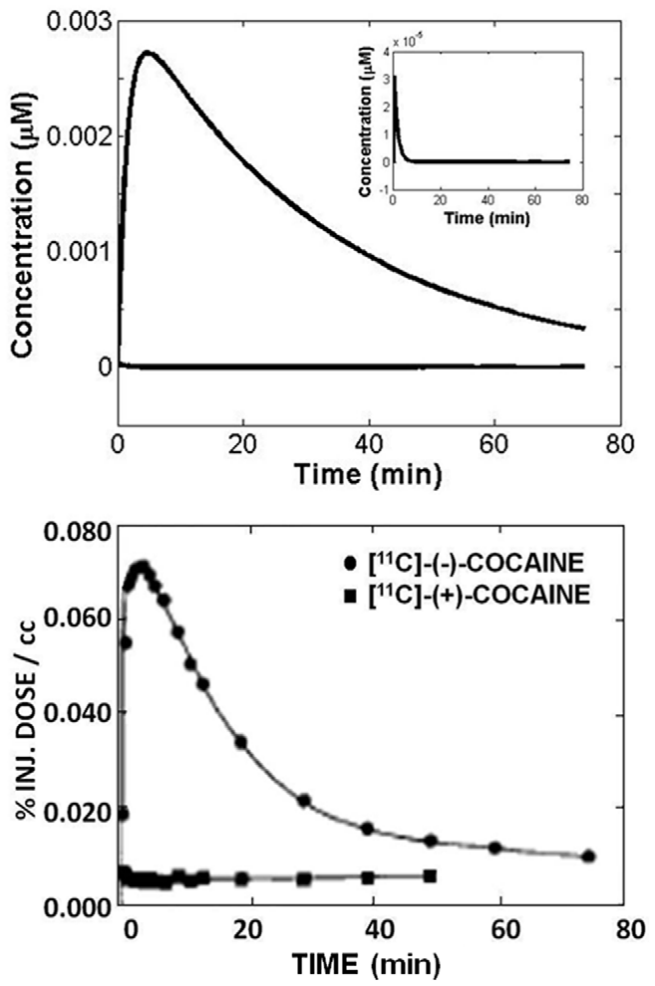
Comparison between the (+)-cocaine hydrolysis by wide-type BChE and the (−)-cocaine hydrolysis by CocH3. Upper panel: modeled (−)-cocaine concentration in the brain of human subject with the presence of wild-type BChE or CocH3 in plasma. Lower panel: reported (−)- and (+)-cocaine concentrations in baboon brain (striatum) with the presence of wild-type BChE in plasma; the data from reference [62].

CocH3 represents a milestone because the catalytic activity of CocH3 against (−)-cocaine (*k*_cat_ = 5,700 min-1 and *K*_M_ = 3.1 µM) is comparable to that of wtBChE against (+)-cocaine. An early study reported by Sun *et al.* [61] determined the catalytic activities of wild-type BChE against both (+)- and (−)-cocaine at the same time. They determined that *k*_cat_ = 6,423±24 min^−1^ and *K*_M_ = 8.5±0.5 µM for wild-type BChE against (+)-cocaine, whereas *k*_cat_ = 3.9±0.8 min^−1^ and *K*_M_ = 9.0±0.3 µM for wild-type BChE against (−)-cocaine. In more recent reports on BChE with (−)-cocaine [21], the catalytic activity of wild-type BChE against (−)-cocaine was characterized more accurately, with *k*_cat_ = 4.1 min^−1^ and *K*_M_ = 4.5 µM. Apparently, the earlier study [61] systematically overestimated the *K*_M_ values for wild-type BChE against both (+)- and (−)-cocaine. To verify this possibility, we have also characterized the catalytic activities of wild-type BChE against (+)- and (−)-cocaine at the same time, indicating that *K*_M_ = 4.7 µM for wild-type BChE against (+)-cocaine while *K*_M_ = 4.5 for wild-type BChE against (−)-cocaine (Yang and Zhan, unpublished results); there was no significant difference between our determined *k*_cat_ values and previously reported *k*_cat_ values. So, the best estimate is that *k*_cat_ = ∼6,423 min^−1^ and *K*_M_ = ∼4.7 µM for wild-type BChE against (+)-cocaine, which is very close to the catalytic activity (*k*_cat_ = 5,700 min^−1^ and *K*_M_ = 3.1 µM) determined for CocH3 against (−)-cocaine. Due to the similar catalytic activities of CocH3 against (−)-cocaine and wild-type BChE against (+)-cocaine, the time course of (−)-cocaine concentration in brain associated with 0.035 µM CocH3 in plasma should be comparable to the corresponding time course of (+)-cocaine concentration in brain associated with the normal 0.035 µM native BChE in plasma (without an exogenous enzyme), with the same 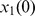 value (corresponding to ∼11 µg) for both (+)- and (−)-cocaine. In fact, the PET data for both [^11^C](+)- and [^11^C](−)-cocaine in baboon (without an exogenous enzyme) were determined under the same experimental conditions by Gatley *et. al.*, as shown in Figure 4 (lower panel) [62]. For a qualitative comparison between the pharmacokinetic modeling for human and the PET data for baboon, the same pharmacokinetic model developed in this study was employed to predict the corresponding time course of (−)-cocaine concentration in human brain associated with 0.035 µM CocH3 in human plasma. The predicted time courses of (−)-cocaine concentrations in human brain with and without 0.035 µM CocH3 are depicted in Figure 4 (upper panel) in comparison with the PET data for the time courses of (−)- and (+)-cocaine concentrations in baboon brain without an exogenous enzyme. As seen in Figure 4, the predicted time course of (−)-cocaine concentration in human brain associated with 0.035 µM CocH3 is indeed very similar to the experimental time course (PET data) of (+)-cocaine concentration in baboon brain, while the predicted time course of (−)-cocaine concentration in human brain without CocH3 agrees with the experimental time course (PET data) of (−)-cocaine concentration in baboon brain. The qualitative agreement between the computational and experimental data depicted in Figure 4 further supports that the model developed in this study is reasonable, giving us additional confidence in the above theoretical evaluations based on the model concerning the perspective of future development of cocaine hydrolase as a therapeutic treatment of cocaine overdose and addiction.

## Discussion

A pharmacokinetic model has been developed to examine the effects of a cocaine-metabolizing enzyme in plasma on the time course of cocaine in plasma and brain of human. The developed pharmacokinetic model has been validated through comparison with available PET data for both (−)- and (+)-cocaine. The modeling has revealed various essentially important, novel insights into cocaine pharmacokinetics in human with and without the presence of an exogenous cocaine-metabolizing enzyme in plasma, including the remarkable differences in the cocaine half-lives and the threshold cocaine concentration or AUC2*_∞_* in brain required to produce physiological effects. It should be pointed out that the current pharmacokinetic model is based on the available PET data in human with only the endogenous enzyme (wtBChE). As it has been well known, there is a significant difference between human and animals in the cocaine pharmacokinetics [37]. It would be extremely difficult to carry out the PET experiments with a therapeutic enzyme candidate and cocaine in human to measure the effects of an exogenous cocaine-metabolizing enzyme on cocaine pharmacokinetics in human. To do such type of PET experiments in human, one must first obtain FDA's approval for using the therapeutic enzyme candidate in human. To file a reasonable Investigational New Drug (IND) application with FDA, one must have compelling reasons concerning why the therapeutic enzyme candidate should work for human. Even if with the FDA's IND approval, one still would have to rationally determine an appropriate, effective concentration of the enzyme for the actual tests in human, because one cannot test an enzyme in human for too many times. So, this pharmacokinetic modeling in human, the endpoint users of our potential anti-cocaine medication, is a crucial step of our enzyme-based therapy development for cocaine abuse. The general insights into the effects of a drug-metabolizing enzyme on drug pharmacokinetics in human should also be valuable for future development of an enzyme therapy for any drug of abuse. The general methodology of the pharmacokinetic modeling may be used to develop valuable pharmacokinetic models for evaluating the effectiveness of metabolic enzymes in detoxifying other drugs.

## Supporting Information

Text S1 Supplementary material available including (1) analysis of the structural identifiability of the model; (2) detailed data for the impacts of the catalytic parameters of enzyme on cocaine concentration in brain; (3) additional information about the evaluation of available high-activity cocaine-metabolizing enzymes for their effects on the cocaine concentration in brain.
(PDF)
