# Supplementary material for: Modeling of Pharmacokinetics of Cocaine in Human Reveals the Feasibility for Development of Enzyme Therapies for Drugs of Abuse
Source: PLoS Comput Biol. 2012 Jul 26;8(7):e1002610. doi: 10.1371/journal.pcbi.1002610 (PMC3406004; doi:10.1371/journal.pcbi.1002610)
Supplement: Text S1 — Supplementary material available including (1) analysis of the structural identifiability of the model; (2) detailed data for the impacts of the catalytic parameters of enzyme on cocaine concentration in brain; (3) additional information about the evaluation of available high-activity cocaine-metabolizing enzymes for their effects on the cocaine concentration in brain. (PDF) [file pcbi.1002610.s001.pdf]

Supplemental Material

Modeling of Chemical Kinetics of Cocaine in Human Reveals the Feasibility for Development of Enzyme Therapies for Drugs of Abuse

Fang Zheng and Chang-Guo Zhan

Department of Pharmaceutical Sciences, College of Pharmacy, University of Kentucky, 789 South Limestone, Lexington, Kentucky 40536

Correspondence:

Chang-Guo Zhan, Ph.D.

Professor

Department of Pharmaceutical Sciences

College of Pharmacy

University of Kentucky

789 South Limestone Street

Lexington, KY 40536

Voice: 859-323-3943

FAX: 859-323-3575

E-mail: zhan@uky.edu

Supplementary material available including (1) analysis of the structural identifiability of the model; (2) detailed data for the impacts of the catalytic parameters of enzyme on cocaine concentration in brain; (3) additional information about the evaluation of available high-activity cocaine-metabolizing enzymes for their effects on the cocaine concentration in brain.

Analysis of the structural identifiability of the model

The system-experiment model is

$$\dot{x}_1(t) = -[V_{\max}/(K_{\mathrm{M}} + x_1(t)) + K_{\mathrm{pb}}]x_1(t) + K_{\mathrm{bp}}x_2(t) \qquad x_1(0) = \frac{D}{V_{\mathrm{p}}} \tag{S1}$$

$$\dot{x}_2(t) = \frac{V_{\mathrm{p}}}{V_{\mathrm{b}}}[K_{\mathrm{pb}}x_1(t) - K_{\mathrm{bp}}x_2(t)] \qquad x_2(0) = 0 \tag{S2}$$

$$y_1(t) = \frac{V_{\mathrm{P}}}{D}x_1(t) \tag{S3}$$

$$y_2(t) = cx_2(t) \tag{S4}$$

$y_1(t)$ and $y_2(t)$ are the model outputs. $V_{\max}$, K_{M}, c, and D (the dose of cocaine) are the known constants, and V_{p}, V_{b}, K_{pb}, and K_{bp} are the technically unknown parameters.

Various methods are available for analysis of the structural identifiability of a model. One of the methods is based on Taylor series expansion of the observations around time t = 0. This method consists of looking at the successive derivatives to check whether they contain information about the parameters to be identified within the model.

The Taylor series expansion of y_i (i = 1 and 2) in t = 0 is

$$y_i(t) = y_i(0) + t\dot{y}_i(0) + \frac{t^2}{2!}\ddot{y}_i(0) + \frac{t^3}{3!}\dddot{y}_i(0) + ... \tag{S5}$$

By using Eqs.(S1) to (S4) and the initial conditions, we have

$$y_1(0) = 1 \tag{S6}$$

$$y_2(0) = 0 \tag{S7}$$

$$\dot{y}_1(0) = -\frac{V_{\max}}{K_{\mathrm{M}} + \frac{D}{V_{\mathrm{p}}}} - K_{\mathrm{pb}} \tag{S8}$$

$$\ddot{y}_1(0) = -[\frac{V_{\max}}{K_{\mathrm{M}} + \frac{D}{V_{\mathrm{p}}}} + K_{\mathrm{pb}}]\dot{y}_1(0) + \frac{V_{\max}\frac{D}{V_{\mathrm{P}}}}{(K_{\mathrm{M}} + \frac{D}{V_{\mathrm{p}}})^2}\dot{y}_1(0) + \frac{V_{\mathrm{P}}K_{\mathrm{bp}}}{Dc}\dot{y}_2(0)$$

or

$$\ddot{y}_1(0) = \dot{y}_1(0)^2 + \frac{V_{\max}\dfrac{D}{V_{\mathrm{P}}}}{(K_{\mathrm{M}} + \dfrac{D}{V_{\mathrm{p}}})^2}\dot{y}_1(0) + \frac{V_{\mathrm{P}}K_{\mathrm{bp}}}{Dc}\dot{y}_2(0) \tag{S9}$$

$$\dot{y}_2(0) = \frac{cDK_{\mathrm{pb}}}{V_{\mathrm{b}}} \tag{S10}$$

$$\ddot{y}_2(0) = \frac{cK_{\mathrm{pb}}D}{V_{\mathrm{b}}}\dot{y}_1(0) - \frac{V_{\mathrm{p}}K_{\mathrm{bp}}}{V_{\mathrm{b}}}\dot{y}_2(0) \tag{S11}$$

…………

$\dot{y}_1(0)$, $\ddot{y}_1(0)$, $\dot{y}_2(0)$, and $\ddot{y}_2(0)$ in Eqs.(S8) to (S11) are all observable parameters. From Eq.(8), we obtain

$$V_{\mathrm{p}} = -\frac{D(K_{\mathrm{pb}} + \dot{y}_1(0))}{K_{\mathrm{M}}K_{\mathrm{pb}} + K_{\mathrm{M}}\dot{y}_1(0) + V_{\max}}\ . \tag{S12}$$

Substitution of Eq.(S12) into Eq.(S9) gives

$$\ddot{y}_1(0) = \dot{y}_1(0)^2 - \frac{\dot{y}_1(0)}{V_{\max}}(\dot{y}_1(0) + K_{\mathrm{pb}})(K_{\mathrm{M}}\dot{y}_1(0) + K_{\mathrm{M}}K_{\mathrm{pb}} + V_{\max}) - \frac{(K_{\mathrm{pb}} + \dot{y}_1(0))K_{\mathrm{bp}}\dot{y}_2(0)}{c(K_{\mathrm{M}}\dot{y}_1(0) + K_{\mathrm{M}}K_{\mathrm{pb}} + V_{\max})}\ . \tag{S13}$$

Equation (S10) gives

$$V_{\mathrm{b}} = \frac{cDK_{\mathrm{pb}}}{\dot{y}_2(0)}\ . \tag{S14}$$

Combination of Eqs.(S10) and (S11) gives

$$\ddot{y}_2(0) = \dot{y}_1(0)\dot{y}_2(0) - \frac{V_{\mathrm{p}}K_{\mathrm{bp}}}{V_{\mathrm{b}}}\dot{y}_2(0)\ . \tag{S15}$$

Substitution of Eqs.(S12) and (S14) into Eq.(S15) gives

$$\ddot{y}_2(0) = \dot{y}_1(0)\dot{y}_2(0) + K_{\mathrm{bp}}\frac{(K_{\mathrm{pb}} + \dot{y}_1(0))\dot{y}_2(0)^2}{(K_{\mathrm{M}}\dot{y}_1(0) + K_{\mathrm{M}}K_{\mathrm{pb}} + V_{\max})cK_{\mathrm{pb}}}$$

or

$$K_{\mathrm{bp}} = \frac{(\ddot{y}_2(0) - \dot{y}_1(0)\dot{y}_2(0))(K_{\mathrm{M}}\dot{y}_1(0) + K_{\mathrm{M}}K_{\mathrm{pb}} + V_{\max})cK_{\mathrm{pb}}}{(K_{\mathrm{pb}} + \dot{y}_1(0))\dot{y}_2(0)^2}\ . \tag{S16}$$

Substitution of Eq.(S16) into Eq.(S13) gives

$$\ddot{y}_1(0) = \dot{y}_1(0)^2 - \frac{\dot{y}_1(0)}{V_{\max}}(\dot{y}_1(0) + K_{\text{pb}})(K_{\text{M}}\dot{y}_1(0) + K_{\text{M}}K_{\text{pb}} + V_{\max}) - \frac{(\ddot{y}_2(0) - \dot{y}_1(0)\dot{y}_2(0))K_{\text{pb}}}{\dot{y}_2(0)}. \quad \text{(S17)}$$

Equation (S17) can be rewritten in the following standard form:

$$A{K_{\text{pb}}}^2 + BK_{\text{pb}} + C = 0 \quad \text{(S18)}$$

in which coefficients *A*, *B*, and *C* can be determined by known constants:

$$A = \dot{y}_1(0)\dot{y}_2(0)K_{\text{M}} \quad \text{(S19)}$$

$$B = 2\dot{y}_1(0)^2\dot{y}_2(0)K_{\text{M}} + \ddot{y}_2(0)V_{\max} \quad \text{(S20)}$$

$$C = \dot{y}_1(0)^3\dot{y}_2(0)K_{\text{M}} + \ddot{y}_1(0)\dot{y}_2(0)V_{\max} \quad \text{(S21)}$$

As well known, Eq.(S18) mathematically can have two solutions, denoted as s_1 and s_2 here for convenience:

$$s_1 = \frac{-B + \sqrt{B^2 - 4AC}}{2A} \quad \text{(S22)}$$

$$s_2 = \frac{-B - \sqrt{B^2 - 4AC}}{2A} \quad \text{(S23)}$$

Further, to know whether s_1 and s_2 are physically meaningful or not, it is interesting to know the signs of the possible *A*, *B*, and *C* values in Eqs.(S22) and (S23). In Eqs.(S19) to (S21), we have known that K_{M} = 4.5 μM and $V_{\max}$ = 4.1 × 0.035 = 0.1435 μM·min^{-1}. Further, $\dot{y}_1(0)$, $\ddot{y}_1(0)$, $\dot{y}_2(0)$, and $\ddot{y}_2(0)$ may be estimated roughly by using the time-dependent cocaine concentrations listed in Table S1, although their exact values are dependent on the specific functions used for the data fitting. Nevertheless, our numerical tests always qualitatively indicate that $A < 0$, $B < 0$, and $C > 0$, which gives

$$-4AC > 0$$

or

$$\sqrt{B^2 - 4AC} > B. \quad \text{(S24)}$$

Equations (S22) to (S24) clearly indicate that $s_1 < 0$ and $s_2 > 0$. For a physically meaningful solution, K_{pb} must be a positive value. Therefore, only one physically meaningful solution can be obtained from Eq.(S18), *i.e.*

$$K_{\text{pb}} = s_2 = \frac{-B - \sqrt{B^2 - 4AC}}{2A}. \quad \text{(S25)}$$

When K_{pb} is known, Eq.(S12) provides V_{p} uniquely, Eq.(S14) provides V_{b} uniquely, and Eq.(S16) provides K_{bp} uniquely.

In summary, although the model mathematically may have two solutions associated with s_1 and s_2, there is only one physically meaningful solution for the values of parameters V_{p}, V_{b}, K_{pb}, and K_{bp} used in the model. So, under the condition that V_{p}, V_{b}, K_{pb}, and K_{bp} all must be positive values, all unknown parameters of the model are uniquely identifiable and, therefore, the model is structurally identifiable.

Impacts of the catalytic parameters of enzyme on cocaine concentration in brain

With all of the model parameters calibrated, we can further discuss possible impacts of the catalytic parameters (k_{cat} and K_{M}) of enzyme on cocaine concentration in brain, *i.e.* $x_2(t)$, when a typical addiction dose of cocaine is administered. It has been known that for a typical addiction dose of cocaine, the peak cocaine concentration in plasma is expected to be about 1 to 5 μM[1,2] Hence, the model was first used to determine the curves of $x_2(t)$ *versus* time (t) in the presence of only endogenous (native) BChE (k_{cat} = 4.1 min^{-1} and K_{M} = 4.5 μM) when $x_1(0)$ = 1 to 5 μM; some important numerical results are summarized in Tables S2 and S3. The obtained curves for $x_1(0)$ = 5 μM are depicted in Figure S2. As seen in Tables S2 and S3, when $x_1(0)$ (which is determined by the dose of cocaine administered) increases, both the peak concentration of cocaine in brain and the AUC2_∞ value increase considerably.

We examined the changes of the cocaine concentration in brain when catalytic parameter k_{cat} or K_{M} of the enzyme is improved, compared to the native enzyme (k_{cat} = 4.1 min^{-1} and K_{M} = 4.5 μM). In particular, we considered the possible change of k_{cat} from 4.1 min^{-1} to 41, 410, 4100, or 41000 min^{-1} when the other parameters (K_{M}, [E], D, V_{b}, V_{p}, K_{pb}, and K_{bp}) are fixed. We also examined the possible change of K_{M} from 4.5 μM to 0.45, 0.045, 0.0045, or 0.00045 μM when the other parameters (k_{cat}, [E], D, V_{b}, V_{p}, K_{pb}, and K_{bp}) are fixed. Important numerical results are summarized in Tables S2 and S3 in comparison with those obtained from the native enzyme. Depicted in Figure S2 are the predicted curves of cocaine uptake and clearance in brain when $x_1(0)$ = 5 μM, corresponding to k_{cat} = 4.1, 41, 410, and 4100 min^{-1} when K_{M} = 4.5 μM (upper panel of Figure S2) and K_{M} = 0.0045, 0.045, 0.45, and 4.5 μM when k_{cat} = 4.1 min^{-1} (lower panel

of Figure S2). As expected, when the enzyme activity increases, the enzymatic hydrolysis of cocaine is always faster so that the $x_2(t)$ value (cocaine concentration in brain) in the presence of a more active enzyme is always smaller than the corresponding $x_2(t)$ value in the presence of only endogenous BChE at any given time (t) after cocaine is administered. The difference between the $x_2(t)$ values corresponding to the two enzymes becomes larger and larger with the time (t), as seen in Figure S2. Hence, when the enzyme activity increases, the cocaine half-life in brain (*i.e.* $t_{b1/2}$), AUC2$_\infty$, and the peak concentration of cocaine in brain all decrease. Due to the decrease of the cocaine peak concentration and half-life in brain, the time to reach the decreased cocaine peak concentration in brain also decreases with increasing the catalytic activity of the enzyme, particularly in increasing k_{cat}, as seen in Tables S2 and S3.

As seen in Table S2 and Figure S2 (upper panel), for a given initial cocaine concentration in plasma, when K_M decreases from 4.5 μM to 0.45 μM, the $t_{b1/2}$ and AUC2$_\infty$ values in brain decrease significantly, while the peak concentration of cocaine in brain also decreases slightly. When K_M decreases further from 0.45 μM, the $t_{b1/2}$, AUC2$_\infty$, and peak concentration of cocaine in brain decrease little. This is because when K_M is sufficiently small, the enzyme has already been saturated and the catalytic reaction has reached the maximum rate such that further decrease in K_M no longer can increase the reaction rate.

As seen in Table S3 and Figure S2 (lower panel), for a given initial cocaine concentration in plasma, when k_{cat} increases for each order of magnitude, the AUC2$_\infty$ value in brain decreases for about a order of magnitude while the $t_{b1/2}$ and peak concentration of cocaine in brain also decrease significantly. Thus, so long as the k_{cat} value is sufficiently large, the $t_{b1/2}$ and AUC2$_\infty$ values and the peak concentration of cocaine in brain all can be neglected.

In addition, because $V_{max} \equiv k_{cat}\cdot[E]$, the same impact of increasing k_{cat} may also be achieved by increasing [E] (the concentration of enzyme). Generally speaking, the higher the enzyme concentration, the greater the maximum reaction velocity (V_{max}) of the catalytic reaction. However, one cannot infinitely increase [E] in practice. For example, in terms of a cocaine hydrolase gene therapy, the enzyme concentration would be subjected to physiological regulation affected by many factors. The actual concentration of an enzyme residing in a living subject might finally reach to an equilibrium value; a BChE mutant gene delivery using a viral vector produced the BChE mutant in rats with a plasma concentration ~0.5 μM for at least a few months.[3]

Additional information about the evaluation of available high-activity cocaine-metabolizing enzymes for their effects on the cocaine concentration in brain

As $AUC2_{\infty}$ and cocaine peak concentration in brain are the primary determinants of the overall cocaine reward/stimulation effects in brain, cocaine-metabolizing enzymes CocHs and CocE all can considerably decrease the overall cocaine reward/stimulation effects in brain. To simplify further discussion below, we will first focus on the $AUC2_{\infty}$ values. As the increase in the initial cocaine concentration in plasma increases both the peak concentration and half-life of cocaine in brain, $AUC2_{\infty}$ is a quadratic polynomial function of $x_1(0)$. Hence, $AUC2_{\infty}$ increases with $x_1(0)$ faster than the simpler linear correction, as the data in Table 1 show.

As seen in Table 1, in the presence of endogenous native BChE with the normal concentration (0.035 μM) in human plasma, $AUC2_{\infty}$ = ~35, ~237, ~10,694, ~39,432, and ~151,039 μM·min when $x_1(0)$ = 1, 5, 50, 100, and 200 μM, respectively. In the presence of 0.035 μM CocE, the respective $AUC2_{\infty}$ values become ~0.07, ~1, ~82, ~321, and ~1,274 μM·min. The respective $AUC2_{\infty}$ values become ~0.005, ~0.07, ~6, ~23, and ~91 μM·min, when the CocE concentration in plasma increases to 0.5 μM. These data suggest that in the presence of CocE (with a reasonable concentration), the $AUC2_{\infty}$ values and, thus, the overall reward/stimulation effects of a typical addiction dose of cocaine corresponding to $x_1(0)$ = 1 to 5 μM are negligible in comparison with the $AUC2_{\infty}$ value of ~35 μM·min associated with 0.035 μM native BChE and $x_1(0)$ value of 1 μM. CocE with a reasonable concentration can be expected to protect the human subjects from the acute toxicity of a lethal dose of cocaine associated with a high $x_1(0)$ value. For example, the $AUC2_{\infty}$ value of ~82 μM·min associated with 0.035 μM CocE and $x_1(0)$ value of 50 μM is close to that associated with 0.035 μM native BChE and $x_1(0)$ value of ~2 μM. The $AUC2_{\infty}$ value of ~321 μM·min associated with 0.035 μM CocE and $x_1(0)$ value of 100 μM is close to that associated with 0.035 μM native BChE and $x_1(0)$ value of ~6 μM. The $AUC2_{\infty}$ value of ~1,274 μM·min associated with 0.035 μM CocE and $x_1(0)$ value of 200 μM is close to that associated with 0.035 μM native BChE and $x_1(0)$ value of ~15 μM. The $AUC2_{\infty}$ value of ~91 μM·min associated with 0.5 μM CocE and $x_1(0)$

value of 200 μM is close to that associated with 0.035 μM native BChE and $x_1(0)$ value of ~2 μM.

As one can see from the data in Table 1, CocHs are more efficient than CocE in decreasing the corresponding $AUC2_\infty$ values under the same dose conditions, because all CocHs have higher catalytic activity than CocE. Within all of these enzymes, CocH3 has the highest catalytic activity and, therefore, CocH3 is most efficient in decreasing the $AUC2_\infty$ value. For example, the $AUC2_\infty$ values associated with 0.035 μM CocH3 when $x_1(0)$ = 1 to 100 μM are all smaller than that associated with 0.035 μM native BChE when $x_1(0)$ = 1 μM. The $AUC2_\infty$ values associated with any CocH (CocH1 or CocH2 or CocH3) of 0.5 μM and any $x_1(0)$ value (1 to 200 μM) examined are all smaller than that associated with 0.035 μM native BChE and $x_1(0)$ value of 1 μM. The relative magnitudes of the calculated $AUC2_\infty$ values associated with CocE and CocH3 are qualitatively consistent with the observed relative potency of these enzymes in the protection of mice from the acute toxicity of a lethal dose of cocaine (180 mg/kg, i.p.). Pharmacokinetic data reported by Pan *et al.*[4] revealed that 30 mg/kg cocaine (i.p.) produced a peak cocaine plasma concentration of 4,100±460 ng/ml or ~14 μM in rat. Based on their pharmacokinetic data, the peak cocaine plasma concentration produced by 180 mg/kg cocaine (i.p.) is estimated to be ~84 μM in the absence of an exogenous enzyme. It has been shown that the minimum effective dose of the exogenous enzyme (i.v.) to fully protect a mouse from a lethal dose of cocaine (180 mg/kg, i.p.) is 0.1 mg for CocE[5] and 0.01 mg for CocH3; CocH3 is ~10-fold more potent than CocE.

It is interesting to compare CocH1 with CocH2 for their relative $AUC2_\infty$ values associated with various $x_1(0)$ values. The $AUC2_\infty$ value associated with CocH2 is smaller than that associated with CocH1 when $x_1(0) < \sim 3$ μM, whereas the $AUC2_\infty$ value associated with CocH1 is smaller than that associated with CocH2 when $x_1(0) > \sim 3$ μM. The change in the relative effects of the enzymes is attributed to the fact that CocH1 (k_{cat} = 3,060 min^{-1} and K_M = 3.1 μM) has a larger catalytic rate constant (k_{cat}, which is the dominant factor affecting the enzyme activity in the condition of a very high substrate concentration), whereas CocH2 (k_{cat} = 1,730 min^{-1} and K_M = 1.1 μM) has a higher catalytic efficiency (k_{cat}/K_M, which is the dominant factor affecting the enzyme activity in the condition of a low substrate concentration).Therefore, it is

significant for improving the potency of the enzyme to decrease K_M when cocaine concentration is low, whereas increasing k_{cat} is always the most effective.

Table S1. The cocaine concentrations in plasma and brain observed by experiment in ref.37 of the text and fitted by the generated kinetic model

Obs. #	Time (min)	Cocaine in brain (Expt. in μM)	$x_2(t)$ (Calc. in μM)	Cocaine in plasma (Expt. in μM)	$x_1(t)$ (Calc. in μM)
1	0.00E+00	0.0E+00	0.0E+00		3.1E-03
2	0.2	1.0E-04	0.4E-03		3.0E-03
3	1.0		1.5E-03	2.9E-3	2.9E-03
4	1.1	1.6E-03	1.6E-03		2.9E-03
5	1.7	2.1E-03	2.1E-03		2.8E-03
6	2.1	2.3E-03	2.3E-03		2.8E-03
7	4.5	2.9E-03	2.7E-03		2.6E-03
8	5.0		2.7E-03	2.7E-3	2.5E-03
9	5.5	2.9E-03	2.7E-03		2.5E-03
10	7.0	2.7E-03	2.6E-03		2.4E-03
11	9.0	2.5E-03	2.5E-03		2.2E-03
12	10	2.4E-03	2.4E-03	2.2E-3	2.2E-03
13	15	2.0E-03	2.1E-03		1.9E-03
14	25	1.5E-03	1.5E-03		1.4E-03
15	30		1.3E-03	1.2E-3	1.2E-03
16	35	1.0E-04	1.1E-03		1.0E-03

Table S2. Effect of K_M on cocaine peak concentration (Peak in μM) and the area under curve ($AUC2_\infty$ in μM·min) in the brain when $k_{cat} = 4.1\ min^{-1}$. Physiological concentration (0.035 μM) of the enzyme in plasma is used in the modeling.

COC (μM)	K_M (μM)									
	4.5		0.45		0.045		0.0045		0.00045	
	Peak (μM·min)	$AUC2_\infty$ (μM·min)	Peak (μM·min)	$AUC2_\infty$ (μM·min)	Peak (μM·min)	$AUC2_\infty$ (μM·min)	Peak (μM·min)	$AUC2_\infty$ (μM·min)	Peak (μM·min)	$AUC2_\infty$ (μM·min)
1	0.908 (5.101)	35.101	0.728 (3.194)	6.963	0.659 (2.688)	3.954	0.649 (2.611)	3.652	0.648 (2.611)	3.622
2	1.842 (5.295)	76.529	1.618 (3.856)	21.143	1.557 (3.545)	15.117	1.550 (3.506)	14.512	1.549 (3.506)	14.452
3	2.795 (5.490)	124.001	2.560 (4.323)	42.538	2.508 (4.089)	33.496	2.502 (4.089)	32.589	2.502 (4.089)	32.499
4	3.762 (5.646)	177.186	3.527 (4.673)	71.149	3.482 (4.517)	59.092	3.477 (4.478)	57.884	3.476 (4.478)	57.763
5	4.738 (5.801)	235.701	4.509 (4.945)	106.978	4.469 (4.828)	91.906	4.465 (4.790)	90.397	4.464 (4.790)	90.246

Table S3. Effect of k_{cat} on cocaine peak concentration (Peak in μM) and the area under curve ($AUC2_\infty$ in μM·min) in the brain when $K_M = 4.5$ μM. Physiological concentration (0.035 μM) of the enzyme in plasma is used in the modeling.

COC (μM)	k_{cat} (min^{-1})									
	4.1		41		410		4100		41000	
	Peak (μM·min)	$AUC2_\infty$ (μM·min)	Peak (μM·min)	$AUC2_\infty$ (μM·min)	Peak (μM·min)	$AUC2_\infty$ (μM·min)	Peak (μM·min)	$AUC2_\infty$ (μM·min)	Peak (μM·min)	$AUC2_\infty$ (μM·min)
1	0.908 (5.101)	35.101	0.560 (2.261)	3.708	0.157 (0.665)	0.371	0.022 (0.129)	0.037	0.002 (0.020)	0.004
2	1.842 (5.295)	76.529	1.178 (2.416)	8.141	0.342 (0.704)	0.816	0.048 (0.133)	0.082	0.005 (0.020)	0.008
3	2.795 (5.490)	124.001	1.842 (2.533)	13.300	0.553 (0.704)	1.335	0.079 (0.137)	0.134	0.008 (0.021)	0.013
4	3.762 (5.646)	177.186	2.544 (2.650)	19.181	0.790 (0.743)	1.927	0.114 (0.140)	0.193	0.012 (0.021)	0.019
5	4.738 (5.801)	235.701	3.278 (2.727)	25.785	1.050 (0.782)	2.593	0.153 (0.144)	0.260	0.016 (0.022)	0.026

Table S4. Lookup table for the predicted cocaine peak concentration, the peak time (Ptime in min), $t_{b1/2}$, and the area under curve in human brain ($AUC2_{\infty}$) with a given initial concentration of cocaine with CocE or CocH or endogenous wtBChE when [E] = 0.5 μM. The K_M values are given in μM, and the k_{cat} values are given in min^{-1}.

COC (μM)	wtBChE (K_M=4.5, k_{cat}= 4.1)		CocE (K_M=0.64, k_{cat}= 468)		CocH1 (K_M=3.1, k_{cat}= 3060)		CocH2 (K_M=1.1, k_{cat}= 1730)		CocH3 (K_M=3.1, k_{cat}= 5700)	
	Peak (Ptime) ($t_{b1/2}$)	$AUC2_{\infty}$ (μM·min)	Peak (Ptime) ($t_{b1/2}$)	$AUC2_{\infty}$ (μM·min)	Peak (Ptime) ($t_{b1/2}$)	$AUC2_{\infty}$ (μM·min)	Peak (Ptime) ($t_{b1/2}$)	$AUC2_{\infty}$ (μM·min)	Peak (Ptime) ($t_{b1/2}$)	$AUC2_{\infty}$ (μM·min)
1	0.488 (1.949) (3.235)	2.596	0.003 (0.020) (1.073)	0.005	0.002 (0.014) (1.080)	0.003	0.001 (0.010) (1.045)	0.002	0.001 (0.008) (1.085)	0.001
5	2.923 (2.348) (3.852)	18.062	0.045 (0.034) (1.059)	0.072	0.012 (0.016) (1.078)	0.020	0.014 (0.013) (1.080)	0.022	0.007 (0.009) (1.084)	0.010
50	43.227 (4.448) (13.267)	748.954	3.416 (0.212) (1.076)	5.827	0.617 (0.041) (1.091)	0.979	1.005 (0.062) (1.071)	1.608	0.334 (0.023) (1.070)	0.526
100	92.703 (5.397) (24.851)	2760.721	12.468 (0.372) (1.110)	22.963	2.301 (0.072) (1.061)	3.698	3.843 (0.116) (1.053)	6.290	1.254 (0.040) (1.093)	1.986
200	193.961 (6.346) (48.900)	10573.310	43.112 (0.665) (1.206)	90.908	8.700 (0.133) (1.077)	14.348	14.524 (0.219) (1.076)	24.857	4.798 (0.073) (1.098)	7.708

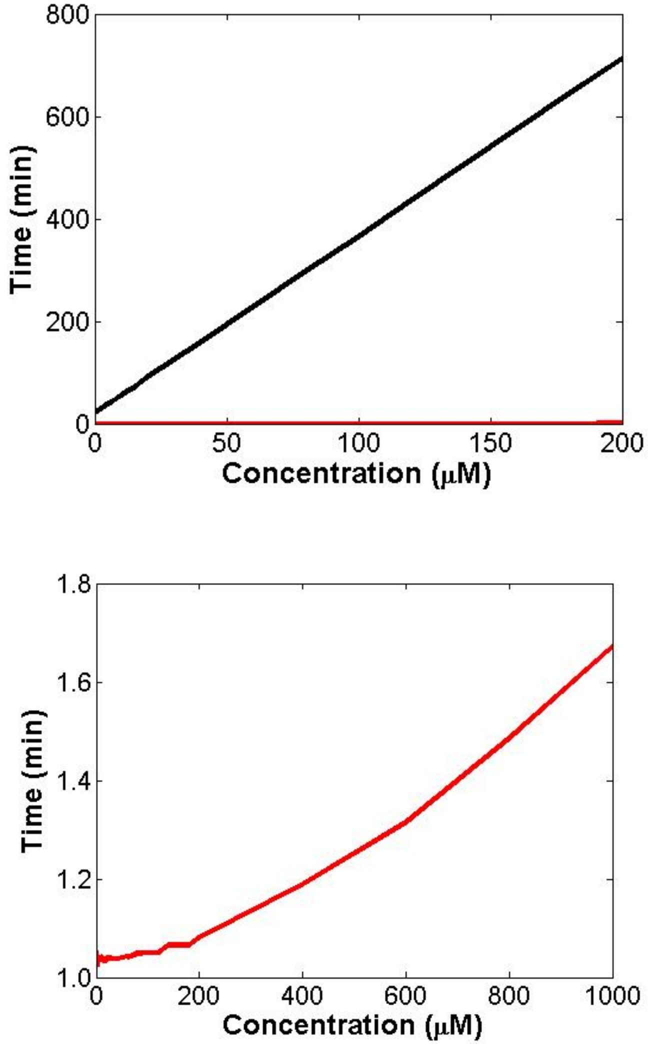

Figure S1. Plots of cocaine half-life in brain ($t_{b1/2}$) *versus* the initial cocaine concentration in plasma ($x_1(0)$). Upper panel (with black line): with only 0.035 μM wtBChE (endogenous BChE, without any exogenous enzyme) in plasma. Lower panel (with red line): in the presence of 0.035 μM CocH3 in plasma.

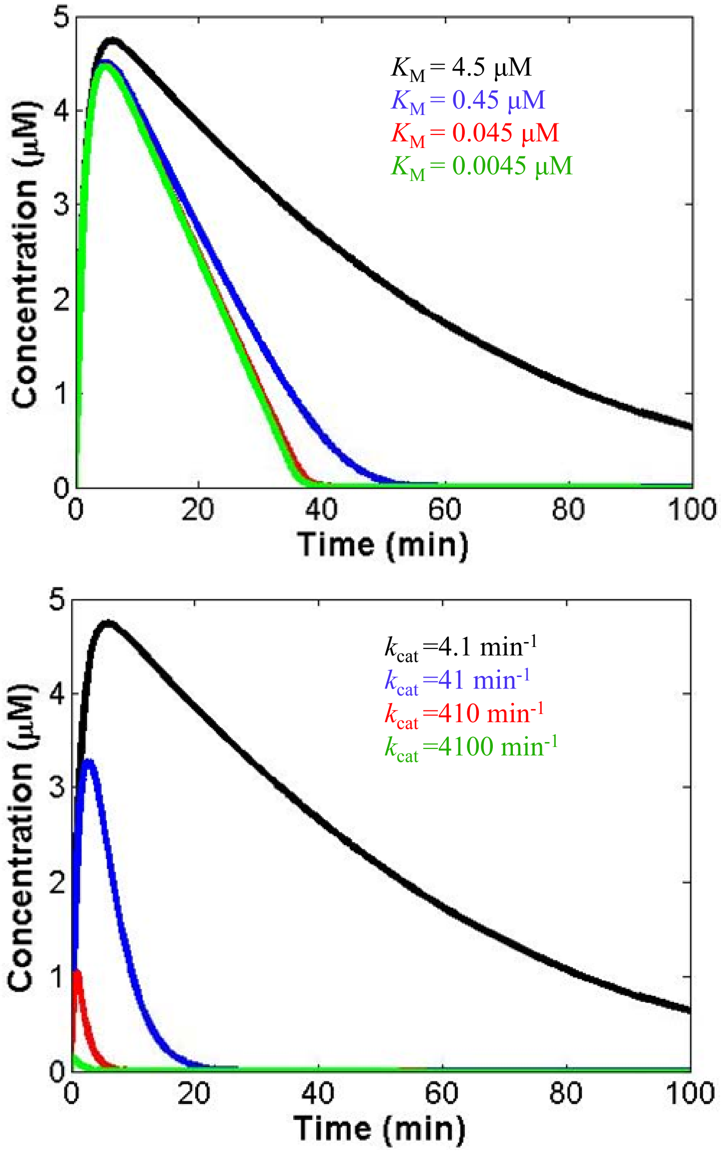

Figure S2. Impact of K_M and k_{cat} of the cocaine-metabolizing enzyme on the cocaine concentration (μM) in brain for a given initial cocaine concentration of 5 μM in plasma. Upper panel: k_{cat} =4.1 min^{-1} with various K_M values. Lower panel: K_M = 4.5 μM with various k_{cat} values.

References

[1] Jufer RA, Walsh SL, Cone EJ (1998) Cocaine and metabolite concentrations in plasma during repeated oral administration: development of a human laboratory model of chronic cocaine use. *J Anal Toxicol* 22: 435-444.

[2] Walsh SL, Stoops WW, Moody DE, Lin SN, Bigelow GE (2009) Repeated dosing with oral cocaine in humans: assessment of direct effects, withdrawal, and pharmacokinetics. *Exp Clin Psychopharmacol* 17: 205-216.

[3] Gao Y, Brimijoin S (2009) Lasting Reduction of Cocaine Action in Neostriatum — A Hydrolase Gene Therapy Approach. *J Pharmacol Exp Ther* 330: 449-457.

[4] Pan WJ, Hedaya MA (1999) Cocaine and Alcohol Interactions in the Rat: Effect on Cocaine Pharmacokinetics and Pharmacodynamics. *J Pharm Sic* 88: 459-467.

[5] Ko M-C, Bowen LD, Narasimhan D, Berlin AA, Lukacs NW, Sunahara RK, Cooper ZD, Woods JH (2007) *J Pharmacol Exp Ther* 320: 926–933.
